# Trained Nurses

**Published:** 1907-09

**Authors:** 


### EDITORIAL NOTES AND COMMENTS.

The Business Office of the JOURNAL-RECORD is 1 1-2 to 5 1-2 South Broad Street.

The Editorial Office is 318-319-320 The Prudential.

Address all Business Commucications to Dr. George Brown, Manager.

Make remittances payable to THE JOURNAI-RECORD OF MEDICINE.

On matters pertaining to the Editorial and Original communications, address Dr. Bernard Wolff, Atlanta.

Reprints of original articles will be furnished at cost price. Requests for the same should always be made on THE MANUSCRIPT.

We will present, postpaid, on request, to each contributor of an original article, twenty (20) marked copies of THE JOURNAL-RECORD OF MEDICINE containing such article.

### TRAINED NURSES.

For several years past, a few ambitious nurses have endeavored to effect some sort of organization and to secure legislation for their protection. Their contention was that the calling of a trained nurse had risen to the dignity of a profession and should be recognized as such. The time, money and effort required to obtain the coveted title of trained nurses were not to be lightly considered and merited for its possessor a position above the Sarey Gamp style of sick-attendant who was a good hand at nursing and fond of it. They maintained that it was not just to them that a nurse without any or very slight preliminary training should be placed on the same footing as concerns importance and preliminary reward as those who had carefully prepared themselves for their work and were in every way competent to perform it. These desires and ambitions have materialized in the passage of the bill creating a Board of Examiners for trained nurses and defining their exact status which has received the governors approval and become a law. Hereafter, all those who desire to engage in the calling of a trained nurse demonstrate their fitness by standing an examination before this board and be accorded a license to carry on their work. The law is not retro-enactive, so that all those who prior to its passage were engaged in this occupation whatsoever be their efficiencies or deficiencies may continue without molestation to administer their sage tea and apply their tar-plasters and peach-leaf poultices as of yore.

The trained nurses are to be heartily congratulated upon the results of their labors for recognition and State support. They have become the chiefest allies and staunchest lieutenants of the doctor and the number of lives that death has been robbed of by their kindly and efficient administration is beyond conjecture and for this alone humanity owes them a debt of gratitude of which recognition of their deserved position in the array of the opponents of disease is but a feeble and insufficient evidence.

